# A national survey of individualized pharmaceutical care practice in Chinese hospitals in 2019

**DOI:** 10.3389/fphar.2023.1022134

**Published:** 2023-03-02

**Authors:** Mingyuan Cai, Liang Zhou, Daihui Gao, Dan Mei, Bo Zhang, Wei Zuo, Qing Yan

**Affiliations:** ^1^ State Key Laboratory of Complex Severe and Rare Diseases, Department of Pharmacy, Peking Union Medical College Hospital, Chinese Academy of Medical Sciences and Peking Union Medical College Hospital, Beijing, China; ^2^ Xiangya School of Pharmaceutical Sciences, Central South University, Changsha, China; ^3^ National Institute of Hospital Administration, Beijing, China

**Keywords:** China, development, individualized pharmaceutical care, pharmacist-management clinic, pharmacogenetic testing, therapeutic drug monitoring

## Abstract

**Background:** Individualized pharmaceutical care, which consists of therapeutic drug monitoring (TDM), pharmacogenetic (PGx) testing and pharmacist-managed clinic (PMC), is one of the most important trends in clinical pharmacy development in the future. While relevant studies in China were primarily single-center or regional. This study aims to explore the current status of individualized pharmaceutical care in China, find out the existing problems and provide references for its further development.

**Methods:** An electronic questionnaire was used and national hospitals’ pharmaceutical administration data from January to December 2019 were collected. The data were sorted into Excel for further statistical analysis. All analyses were descriptive.

**Results:** The proportions of hospitals that performed TDM and PGx testing were 12.83% and 9.48%, respectively. The major responsible departments were the clinical laboratory and pharmacy department. External quality control was carried out in around 70% of hospitals for both TDM and PGx testing. More than half of hospitals provided TDM services for valproate sodium, digoxin, carbamazepine, vancomycin and cyclosporine. And an average of 6.84 drugs were performed TDM in 540 hospitals. Clopidogrel and warfarin were the top two drugs that performed PGx testing. As for the PMC, 10.03% of hospitals opened PMC, of which 60.00% had independent PMC. Approximately 80% of PMC services were free of charge.

**Conclusion:** The development of individualized pharmaceutical care in China is still in the early stage. Different sectors have to coalesce to promote its implementation, including the appropriate education, coverage, reimbursement policies, high-quality evidence, data systems, health system processes and health policies, *etc.*

## 1 Introduction

Considerable attention has been paid to “precision medicine” in recent years. It refers to treatments using individuals’ genetic, biomarker, phenotypic, or psychosocial characteristics to distinguish a specific patient from others with similar clinical manifestations (Jameson and Longo, 2015). Individualized pharmaceutical care is a component of precision medicine that mainly aims at individualized pharmacotherapy. Therapeutic drug monitoring (TDM) and pharmacogenetic (PGx) testing are two essential approaches to making scientific drug regimens based on individual differences among patients. Meanwhile, opening a pharmacist-managed clinic (PMC), or a medication therapy management clinic (MTMC) is another significant step toward giving patients individualized pharmaceutical treatment. The implementation of TDM, PGx testing and PMC are all important references to evaluate the quality of individualized pharmaceutical care.

Having an overall grasp of individualized pharmaceutical care is conducive to identifying the existing problems and better promoting its further development. However, many recent studies concerning TDM, PGx or PMC in China were single-center or regional in nature (Zhang C. et al., 2021; Zhang J. et al., 2021; Liu et al., 2021). Some studies have explored healthcare providers’ and researchers’ understanding of PGx and factors that hindered the PGx clinical application (Guo et al., 2021), but the overall situation of individualized pharmaceutical care in China (including the implementation rate, responsible departments, the conduction of quality control and so on) is still unknown. We thus perform this national questionnaire survey regarding TDM, PGx and PMC in clinical practice to comprehensively know about the current situation of individualized pharmaceutical care in China and raise potential solutions to existing problems, hoping to promote the long-term, healthy growth of this field.

## 2 Materials and methods

### 2.1 Data collection

**FIGURE 1:**
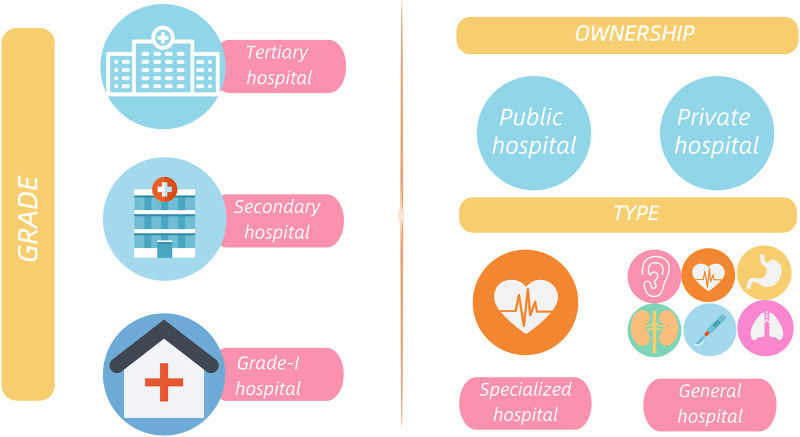
The grade, ownership and type of hospitals in China, Grade of hospitals: According to the *Hospital Grading Management Standard*, Chinese hospitals are divided into 3 grades, i.e., tertiary, secondary and grade-I hospitals, based on a series of indicators (hospital scale, research capabilities, medical facilities, *etc.*). Among these grades, the tertiary hospital is the premier. Ownership of hospitals: Public hospitals are hospitals funded by the country and taken into the government’s financial budget. Being the mainstay of Chinese medical service system, public hospitals are directly managed by local health authorities, emphasizing public welfare. Private hospitals refer to health institutions funded by societies and dominated by for-profit institutions. The establishment of private hospitals must be approved by local health authorities. Type of hospitals: includes general and specialized hospitals.

This study was nested within a nationwide survey, undertaken by the National Institute of Hospital Administration to collect data on pharmaceutical administration and quality control in medical institutions. Chinese hospitals are rated in 3 grades based on a series of indicators (hospital scale, research capabilities, medical facilities, etc.). The top grade among these is the tertiary hospital. Given the difficulty of collecting data from grade-I hospitals, our target hospitals were confined to secondary and above general hospitals, and the ownership had no limitation (Figure 1 introduced the grade, ownership and type of hospitals in China). Heads of medical affairs departments in these hospitals were requested to register and log on to the accompanying website National Clinical Improvement System (National Clinical Improvement System, 2019) to fill out questionnaires concerning pharmaceutical administration, including individualized pharmaceuticals care. The guideline for data collection was provided in (Supplementary material S1). The collected data ranged from January 2019 to December 2019. To ensure the accuracy and reliability of the data, each hospital was requested to designate a responsible person to check the reported data. Additionally, the questionnaire’s data quality will serve as a benchmark for hospital evaluation and key specialty settings. This survey was approved and organized by the National Institute of Hospital Administration in the national layer and was approved by the Ethical Committee of the Peking Union Medical College and Chinese Academy of Medical Sciences (Beijing, China).

### 2.2 Questionnaire development

The questionnaire was designed by experts from the National Institute of Hospital Administration, covering a wide range of items concerning pharmaceutical management and quality control. Two rounds of Delphi survey through online questionnaire were conducted and 105 expert opinions were collected in total. The survey was modified based on the results of expert opinions. A consensus was reached after an experts’ face-to-face discussion. In this paper, we focus on items related to individualized pharmaceutical care. Concerning questions were excerpt from the questionnaire of Medical Quality Management and Control in 2019 (Supplementary material S2). These questions can be divided into five parts, (1) basic hospital information; (2) TDM implementation situation; (3) PGx testing implementation situation; (4) the situation of PMC and (5) supplementary information. Explanations for specific notions (such as PGx testing) have been given in the questionnaire and the hotline was available for any inquiries.

### 2.3 Data analyses

The data collected from the network survey was sorted into Microsoft Excel 2016 for further statistical analysis and chart drawing. We analyzed the data as a single cohort or stratified by grade and ownership of hospitals. Indexes analyzed in our survey were summarized in Table 1. The indices of hospitals with TDM/PGx/PMC and the conduction of external quality control were analyzed by percentage. On account of the indexes of drugs (or genes) tested in TDM and PGx testing services, the content filled in by different hospitals varies greatly because of the fill-in-the-blank form, adding difficulties for further analysis. To make data standardized for further analysis, we first sorted out hospitals with valid information, then extracted the data according to the kind of drugs (or genes) and counted the total number of drugs (or genes) for each hospital. In the specific analysis, we excluded hospitals in which data was missing or deviated from reality. All of our analyses were descriptive.

**TABLE 1:** Indexes analyzed in our survey.

Section	Evaluation Indexes	Details
TDM	Hospitals with TDM (%)	The number of hospitals implementing TDM/Total number of hospitals with valid data
	Responsible department	Pharmacy department; Clinical laboratory; Others
	EQC*^a^* (%)	The number of hospitals conducting EQC for TDM/Total number of hospitals with valid data
	Drugs tested	All hospitals filled in this index according to the actual situation in their own hospitals
PGx	Hospitals with PGx testing (%)	The number of hospitals implementing PGx testing/Total number of hospitals with valid data
	Responsible department	Pathology department; Clinical laboratory; Pharmacy department; Multi-department; others
	EQC (%)	The number of hospitals conducting EQC for PGx testing/Total number of hospitals with valid data
	Drugs and genes tested	All hospitals filled in this index according to the actual situation in their own hospitals
PMC/MTMC	Hospitals with PMC (%)	The number of hospitals providing PMC/Total number of hospitals with valid data
	Types*^b^*	Collaborative PMC; Independent PMC (Specialized/General)
	Charge	Yes or No

TDM: Therapeutic drug monitoring; PGx: Pharmacogenomics; PMC: Pharmacist-managed clinic; MTMC: Medication therapy management clinic; EQC: External Quality Control, or External Quality Assessment (EQA).

^a^ External quality control is organized by the clinical laboratory center (or reference laboratory) under the leadership of the health department, to ensure the reliability of the testing results in hospital laboratories. The reference laboratory delivers quality control samples to hospital laboratories and requires them to report the testing results in a limited time. Then reference laboratory will analyze the submitted results to find out existing problems, eventually improving the testing abilities in hospital laboratories.

^b^ Collaborative PMC, refers to a service mode in which pharmacists provide pharmaceutical care for patients together with doctors or personnel from other departments; Independent PMC, refers to a service mode in which pharmacists provide pharmaceutical care independently. Independent PMC, includes specialized clinics and general clinics.

## 3 Results

Overall, we delivered electronic questionnaires to 4 750 hospitals nationwide, and the total numbers of respondents were 4 637 for TDM, 4 640 for PGx and 4 638 for PMC, respectively.

### 3.1 Status of therapeutic drug monitoring

#### 3.1.1 Hospitals with TDM

In total, 4 637 hospitals with valid data were included in this item. Of these, 595 (12.83%) hospitals carried out TDM service, the number and percentage of hospitals with TDM classified by grade and ownership were shown in Table 2. The percentage of hospitals with TMD ranged from 35.28% (in tertiary public hospitals) to 1.70% (in secondary private hospitals), which indicated that the clinic implementation of TDM was not widespread throughout the country. The percentage of tertiary hospitals with TDM was higher than secondary hospitals, and the public was higher than private.

**TABLE 2:** The percentage of hospitals with TMD, PGx testing and PMC.

Hospitals	Hospitals with TMD%	Hospitals with PGx testing%	Hospitals with PMC%
Tertiary Public hospitals	35.28 (471/1 335)	27.02 (361/1 336)	24.70 (330/1 336)
Secondary Public hospitals	3.84 (96/2 502)	2.24 (56/2 503)	4.44 (111/2 502)
Tertiary Private hospitals	17.20 (16/93)	12.90 (12/93)	6.45 (6/93)
Secondary Private hospitals	1.70 (12/707)	1.55 (11/708)	2.55 (18/707)
All hospitals	12.83 (595/4 637)	9.48 (440/4640)	10.03 (465/4638)

#### 3.1.2 Responsible department

**FIGURE 2:**
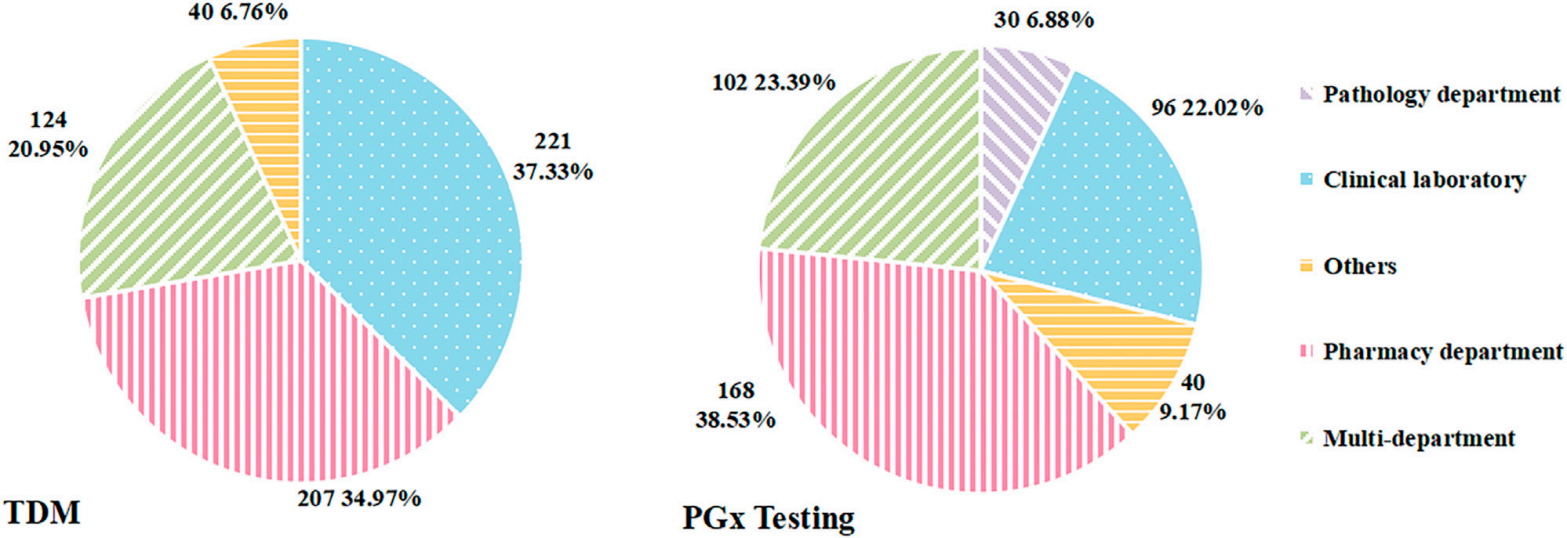
The proportion of different departments in charge of TDM and PGx testing.

As for the department in charge of TDM service, three of the 595 hospitals that carried out TDM were excluded from further analyses for data deficiency. As shown in Figure 2, the major departments in charge of TDM were the clinical laboratory and pharmacy department. TDM was conducted independently by the clinical laboratory in 221 hospitals (37.33%), followed by the pharmacy department in 207 hospitals (34.97%).

#### 3.1.3 External quality control for TDM

External quality control (EQC), also called external quality assessment (EQA), is a crucial part of clinical laboratory quality management. It is organized by the Clinical Laboratory Center (or Reference Laboratory) under the leadership of the Health Department, to ensure the reliability of the testing results in hospital laboratories. The reference laboratory delivers quality control samples to hospital laboratories and requires them to report the testing results in a limited time. Then the reference laboratory will analyze the submitted results to find out existing problems, eventually improving the testing abilities in hospital laboratories. Of 595 hospitals that provided TDM service, 412 (69.24%) conducted external quality control (EQC) for TDM. Then we stratified the hospitals by grade and ownership. It showed that the proportions in tertiary public, secondary public, tertiary private and secondary private hospitals were 73.04% (344/471), 57.29% (55/96), 56.25% (9/16) and 33.33% (4/12), respectively.

#### 3.1.4 Drugs tested

**FIGURE 3:**
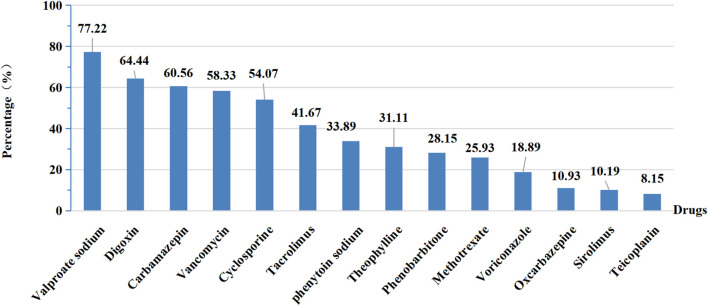
The percentage of drugs performed TDM in 540 hospitals.

Of 595 hospitals, only 540 offered valid information about specific drugs carried out TDM. We firstly sorted out all kinds of drugs for TDM from the provided information and then counted the total number of drugs for each hospital. As shown in Figure 3, more than half of hospitals provided TDM services for valproate sodium, digoxin, carbamazepine, vancomycin and cyclosporine. And an average of 6.84 drugs were performed TDM in 540 hospitals. After we stratified these hospitals by grade and ownership, it revealed that tertiary public hospitals performed 7.43 drugs for TDM on average, which was the highest among these four types of hospitals (Table 3).

**TABLE 3:** Drugs performed TDM on average in 540 hospitals.

Hospitals	Total	Drugs performed TDM on average
	N*	
Tertiary Public hospitals	448	7.43
Secondary Public hospitals	73	3.81
Tertiary Private hospitals	12	5.71
Secondary Private hospitals	7	3.29
All hospitals	540	6.84

N*: indicates the number of respondents provided valid information.

### 3.2 Status of pharmacogenetic testing

#### 3.2.1 Hospitals with PGx testing

Of 4 640 hospitals with available data, 440 (9.48%) implemented PGx testing. Table 2 showed the number and proportion of hospitals with PGx testing stratified by grade and ownership. It also presented that the proportion of tertiary hospitals with PGx testing was higher than that of secondary hospitals. Furthermore, the proportion was higher in public hospitals than that in private hospitals.

#### 3.2.2 Responsible department

After excluding four hospitals with missing data of this index, the number of hospitals with valid data was 436. Of these, PGx testing in 168 hospitals was undertaken by the pharmacy department, accounting for the largest proportion (38.53%), followed by multi-department in 102 (23.39%) hospitals (Figure 2).

#### 3.2.3 EQC for PGx testing

Among the 440 hospitals with PGx testing, 309 (70.23%) conducted EQC for PGx testing. And the proportions in tertiary public, secondary public, tertiary private and secondary private hospitals were 72.00% (260/361), 66.07% (37/56), 58.33% (7/12) and 45.45% (5/11), respectively.

#### 3.2.4 Drugs and genes tested

**FIGURE 4:**
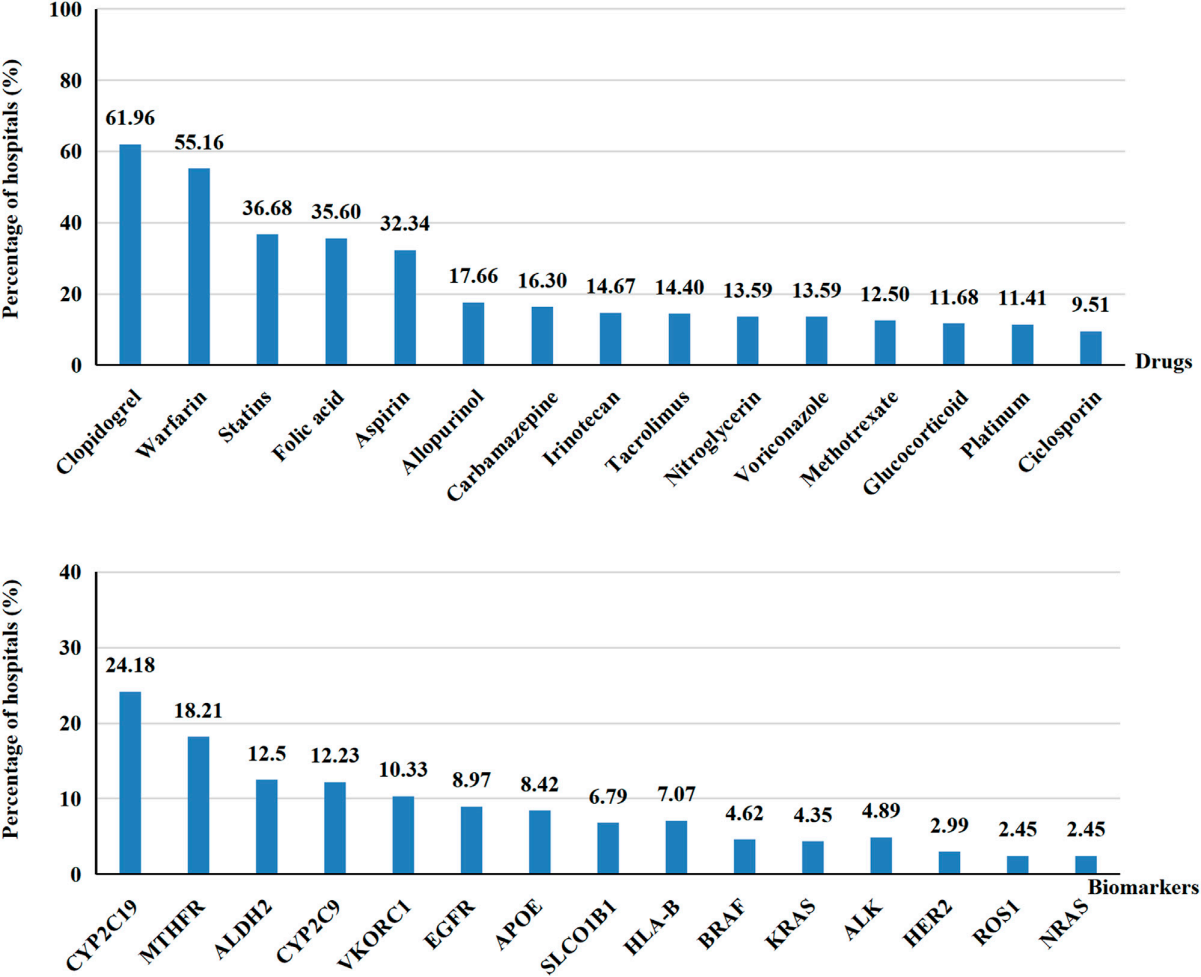
The percentage of drugs and genes performed PGx testing.

Most hospitals filled this index with drugs and/or genes, but a few hospitals filled it with disease or irrelevant information, making it impossible to determine whether a specific drug performed PGx testing in those hospitals. We therefore excluded these hospitals to ensure the accuracy of our results. Ultimately, a total of 368 hospitals were included. As shown in Figure 4, we ranked drugs and genes by the number of hospitals offering PGx testing for them. Clopidogrel, warfarin, statins, folic acid and aspirin were the top five drugs that performed PGx testing in 368 hospitals. As for genes, the top five were the cytochrome P450 (*CYP*) *2C19* gene, 5,10-methylenetetrahydrofolate reductase (*MTHFR*), Aldehyde dehydrogenase 2 (*ALDH2*), *CYP2C9* and Vitamin K epoxide reductase complex subunit 1 (*VKORC1*).

### 3.3 Status of pharmacist-managed clinic

#### 3.3.1 Hospitals with PMC

A total of 4 638 hospitals were included in this index, among these, 465 (10.03%) hospitals set up PMC. From Table 2 we can see that the proportion of hospitals with PMC varied by grade and ownership.

#### 3.3.2 Types of PMC

**FIGURE 5:**
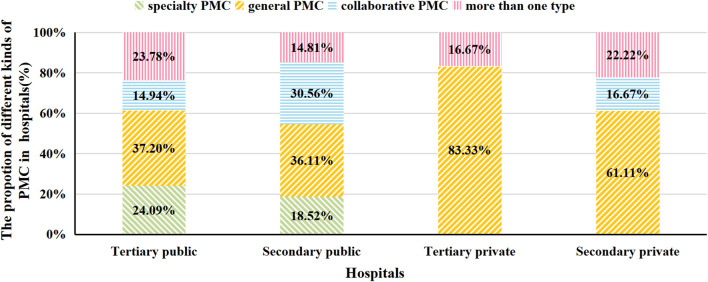
The proportion of different kinds of PMC in hospitals.

We excluded five hospitals that did not fill in this index. Among 460 hospitals, 85 (18.48%) hospitals had collaborative PMC, 276 (60.00%) had independent PMC, including 99 (21.52%) specialized and 177 (38.48%) general clinics. Besides, 99 (21.52%) hospitals had more than one type of PMC. When these hospitals were stratified by grade and ownership, we found that in all kinds of hospitals, general PMC accounted for the largest share (Figure 5).

#### 3.3.3 Charge

In this index, all hospitals provided valid data. Of 465 hospitals with PMC, only 98 (21.08%) were charged. PMC in six tertiary private hospitals were all free of charge. And the proportions of charging PMC in tertiary public hospitals, secondary public hospitals and secondary private hospitals were 27.27%, 5.41% and 11.11%, respectively.

## 4 Discussion

Our study intends to investigate the state of individualized pharmaceutical care in China, find out any unsolved issues, and offer solutions for its further advancement. We found that the implementation rate of TDM, PGx and PMC is relatively low (12.83%, 9.48% and 10.03%, respectively). The responsible departments of TDM and PGx testing among hospitals are not unified yet, and the clinical laboratory and pharmacy department were the major responsible departments. About 70% of hospitals conducted EQC for TDM, and the proportion is similar for PGx testing. TDM services for valproate sodium, digoxin, carbamazepine, vancomycin, and cyclosporine were offered by more than 50% of hospitals. Clopidogrel and warfarin were the top two drugs that performed PGx testing. Approximately 80% of PMC services were free of charge. Based on the results of this survey, we discussed the proportion of hospitals with individualized pharmaceutical care, responsible departments, the conduction of quality control, specific implementing projects for TDM/PGx and the development of PMC in turn.

### 4.1 The proportion of hospitals with individualized pharmaceutical care

Our survey provides fresh reference regarding the current situation of individualized pharmaceutical care all over China. In general, tertiary hospitals had higher proportions of hospitals with TDM, PGx testing, or PMC services than secondary hospitals, and public hospitals had higher proportions than private hospitals, reflecting the unbalanced development situation among hospitals. Besides, there is still a big gap between China and the United States in the implementation of TDM. According to the American Society of Health-System Pharmacists (ASHP) national survey findings in 2015, 70.6% of hospitals used TDM to routinely monitor patients for adverse events (Pedersen et al., 2016), which was around two-fold the rate in our tertiary public hospitals (35.28%). However, it is noteworthy that multiple metrics from different dimensions need to be measured to comprehensively assess the advance of individualized pharmaceutical care, including the implementing rate of TDM, PGx or PMC, the conduction of quality control, the number of drugs performed TDM or PGx, patient satisfaction, and so on. It cannot be inferred merely from the implementing rate of TDM. Quantitative methods are needed to evaluate the quality of pharmaceutical care more scientifically and roundly.

Interestingly, we discovered that the proportions of hospitals with TDM were even lower than the proportions from older studies in China. A study conducted by Chen et al., in 2013 reported that 89.89% (80/89) hospitals had provided TDM service (Chen et al., 2015), and another survey conducted by Zhang et al., in 2019 showed that 75.86% (22/29) hospitals offered TDM service (Zhang C. et al., 2021), which was much higher than our results in 2019. We speculated that the excessively high rates in these studies were mainly caused by the inclusion of unrepresentative hospitals. Chen et al. selected 89 public hospitals as objects, and Zhang et al. only included 29 tertiary public hospitals in northern China. Moreover, results can also be influenced by the attitude of staff filling out the questionnaire. The data Chen et al. used were submitted by hospitals to evaluate key specialty departments, thus data providers were more likely to show their situation in an overly flattering light. On the contrary, our survey was carried out for the aim of pharmaceutical quality control and had nothing to do with the hospital evaluation.

As a whole, our results indicated that the popularization of TDM, PGx and PMC in China is at a relatively low level. However, in recent years, the Chinese authorities strongly support the implementation of pharmaceutical care. In 2018, the National Health Commission (NHC) and the National Administration of Traditional Chinese Medicine promulgated *Opinions on accelerating the high-quality development of pharmaceutical care*, emphasizing the importance of pharmaceutical care for people’s health (National Health Commission, 2018). Two transformations for the mode of pharmaceutical care are proposed: shifts from “drug-centric” to “patient-centric”; and from “focusing on ensuring drug supply” to “focusing on providing professional pharmaceutical services and participating in clinical practice of drug use on the premise of ensuring drug supply”. To further promote and standardize pharmaceutical care, the NHC has formulated service specifications for pharmacist-managed clinics, drug use education and pharmaceutical monitoring service in medical institutions in 2021 (National Health Commission, 2021). These documents all reflect that the Chinese authorities have attached great importance to pharmaceutical care. On account of lacking longitudinal data about TDM, PGx and PMC, it was impossible to analyze the impact of policies on the implementation of individualized pharmaceutical care. We will pay close attention to its development tendency and discuss it in the follow-up research.

### 4.2 The situation of responsible departments of TDM and PGx testing

Our results showed that the responsible departments for TDM and PGx varied greatly among Chinese hospitals. The responsible departments are largely concentrated in the pharmacy department, clinical laboratory and multi-department. The different educational backgrounds among these departments contribute to the discrepancy in clinical practice. Practitioners in the clinical laboratories tend to stress the operation standards and internal or external quality control, while pharmacists are more likely to focus on pharmacokinetics and the adjustment of drug regimens according to the interpretation of testing results. The diversity of responsible departments inevitably increases the difficulty of standardization and conducting quality control for clinical practice.

The responsible departments need to be standardized urgently. In China, guidelines for the management of TDM practice have not yet been published. Only *the Expert Consensus on the Standards of Therapeutic Drug Monitoring* was published in 2019 (Zhang et al., 2019), putting forward that the qualification certification for TDM professionals ought to be conducted, but the detailed information about responsible departments was not mentioned. Given that TDM is a multidisciplinary field, one possible strategy is that different departments specialized in different fields took corresponding responsibilities in TDM procedure. Physicians could submit test requests in clinical practice; sample collection and concentration determination could be conducted by professionals in clinical laboratories; clinical pharmacists could be responsible for data processing and interpretation of results for patients. Through the collaboration of multiple departments and a clear division of responsibility, the management of clinical practice will be standardized and the working efficiency will be improved.

**FIGURE 6:**
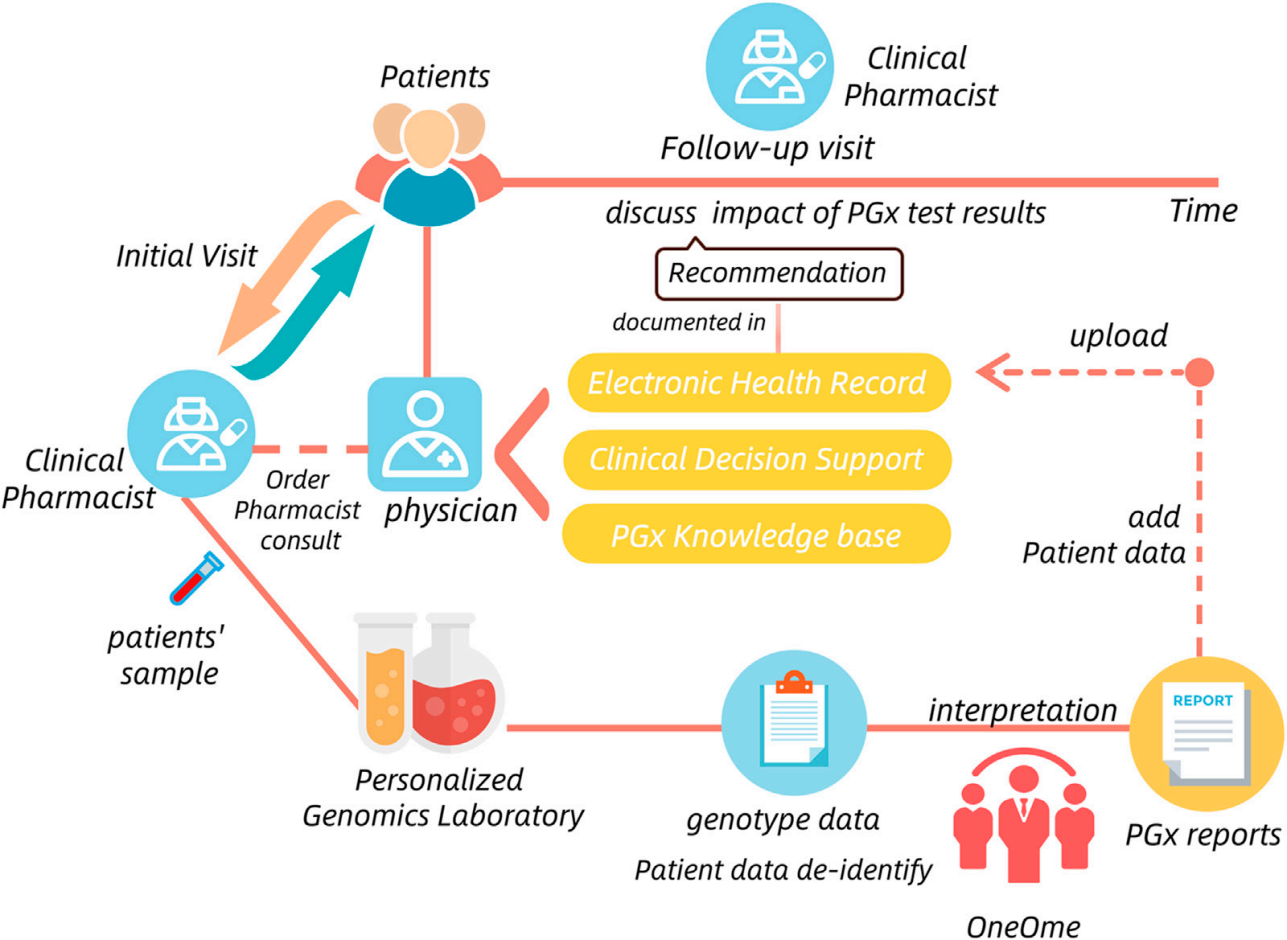
The process and involved departments of PGx Testing in the Mayo Clinic.

The situation of PGx testing was similar to TDM. Four published guidelines concerning the management of PGx testing only stressed the importance and necessity of PGx-staff qualification certification, the detailed information for responsible departments was also omited (National Health and Family Planning Commission, 2015). Fortunately, many pilot hospitals where PGx testing has been successfully implemented provide us with good models to learn from. For example, the Mayo Clinic had implemented the Nine-gene pharmacogenomics profile service, which was a pre-emptive PGx testing process. It established a care model including the Center for Individualized Medicine and pharmacy operations teams, clinical decision support team, Personalized Genomics Laboratory and OneOme (a commercial vendor) that provided reports of PGx results (Matey et al., 2022) (Figure 6). In China, Beijing Chaoyang hospital also conducted PGx testing in the charge of clinical pharmacists and pharmacogenetic technicians. They all show that the clinical practice of PGx testing usually involves multiple departments and institutions.

Additionally, although TDM and PGx testing in most of hospitals are multi-sectoral participation, a backbone is still needed. Given that clinical pharmacists work on the front line with patients and have a solid foundation of pharmacy knowledge, they are theoretically well-suited to promote the application of both TDM and PGx testing in clinical practice. It is suggested that the clinical pharmacy department should play a leading role in these related departments and coordinate the clinical implementation of TDM and PGx testing on the whole.

However, most of clinical pharmacists today are unable to interpret the TDM and PGx testing results professionally, especially for PGx testing. Zhang et al. reported that the majority of pharmacists in the First Affiliated Hospital of Zhengzhou University had no or little knowledge about the PGx because of the absence of PGx education in their major during the master or doctor school year (Zhang J. et al., 2021). Hu et al. also reported that approximately 60% of pharmacists believed they had poor or fair capacity to offer PGx testing and related services (Hu et al., 2022). In Japan, only 12.4% of pharmacists had received specific PGx-related education (Tsuji et al., 2021). Therefore, the educational system should be established as soon as possible. Post-graduate opportunities, such as easily accessing continuing education courses and Graduate certifications or Master’s Programs could be provided for pharmacists; colleges of pharmacy may offer courses focused on TDM and PGx in their curriculum as part of pharmacology curricula or as an elective course for students (Kisor et al., 2018; Marcinak et al., 2018; Mayo Clinic, 2019; University of Florida Health, 2022).

### 4.3 The conduction of quality control for TDM and PGx testing

Conducting quality control is important to ensure the reliability of the TDM and PGx testing results in hospital laboratories, which is the prerequisite for guiding patients to use drugs rationally. EQC plays a significant role in the quality control system. Its primary goal is to determine whether testing systematic mistakes exist in laboratories’ clinical practice and identify the sources of errors so as to enhance the reliability of testing findings and advance laboratory analysis technology. In our survey, the proportions of hospitals conducting EQC for TDM and PGx were comparable to each other (69.24% and 70.23%, respectively). There is also obtained information regarding quality control or quality assessment in other countries. In South Korea, 20%–29% of respondents did not participate in proficiency testing for EQC in 2018 (Choi et al., 2019), which was roughly the same percentage as in Chinese hospitals. Similarly, in Malaysia, 77.4% of hospitals reported having quality assurance programs for TDM in their hospitals (such as international quality assessment programs, and the hospital’s internal quality assessment programs) (Ab Rahman et al., 2013).

In the United States, to guarantee the reliability of testing results, laboratories performing PGx testing are subject to federal regulatory standards through College of American Pathologists accreditation and Clinical Laboratory Improvement Amendment certification or stricter state requirements (Vo et al., 2017). In China, EQA is routinely organized by pharmaceutical quality control institutions, clinical laboratory centers or other pharmaceutical societies approved by national/provincial/municipal health commissions (Division of Therapeutic Drug Monitoring et al., 2021). In general, the National Center for Clinical Laboratories organizes quality control of clinical laboratories nationwide, mainly for large medical institutions (National Center for Clinical Laboratories, 2022), while the provincial clinical laboratory centers are responsible for quality control of local clinical laboratories, primarily in regional, city, county or some township medical institutions (Wenxiang, 2013). Typically, quality control institutions will specify a catalogue of projects that they are qualified for conducting EQA, and laboratories can choose from the catalogue according to the projects conducted in their own laboratories (National Center for clinical Laboratories et al., 2013). However, in certain situations, projects implemented in laboratories are not all included in the catalogue. That is to say, the diversity of testing projects and methods is one of the reasons that hinders the conduction of EQC for TDM and PGx testing. It is recommended that quality control institutions build connections with corresponding laboratories, collect up-to-date testing projects from subordinate laboratories regularly, and then develop standard operation procedures (SOP) accordingly. For testing projects that have no SOP of EQA currently, other laboratories conducting the same or related projects could be references.

Our survey collected information about the conduction of EQC for TDM and PGx testing, which can reflect the testing abilities of laboratories but cannot point out the testing stability (or precision) for a specific testing project in clinical practice. Only through conducting internal quality control can we discover and remedy existing problems in time to ensure the consistency of testing results for patients. In addition, other processes such as sample collection, transportation and reporting of test results all require quality control. Further investigations about quality control remain to be conducted.

### 4.4 Analysis for implementing projects of TDM and PGx testing

In general, the drugs performed TDM and PGx testing in our survey were consistent with other contemporary small-scale studies in China (Zhang C. et al., 2021; Zhang J. et al., 2021). The results indicated that over 50% of hospitals performed TDM for valproate sodium, digoxin, carbamazepine, vancomycin and cyclosporine. Of note, most of these drugs have narrow therapeutic ranges and high intra- and inter-individual variability (Patsalos et al., 2018; He et al., 2020; Deprez and Stove, 2021; Ballotari et al., 2022). In these scenarios, TDM is useful to adjust medication dosages of treatments to maximize clinical effectiveness and decrease their toxicity. Situations that require TDM to guide medication are summarized in Table 4 (Muller et al., 2018; Alrabiah et al., 2021).

**TABLE 4:** Scenarios that require TDM.

Types of scenarios	Scenarios
Drug-related scenarios	drugs with narrow therapeutic ranges
	drugs that have non-linear pharmacokinetics
	drugs that interindividual pharmacokinetics vary greatly
	antibiotic drugs that need to prevent drug resistance
Clinical practice-related scenarios	requiring long-term medication but lacking a clear, observable endpoint or indicator of treatment
	to seek causes of therapeutic failure
	the toxicity of drug therapy is difficult to distinguish from the patient’s underlying disease
Patient-related scenarios	patients’ clearance ability of drugs with narrow therapeutic indexes has been impaired
	to monitor patient’s compliance
	for patients with social habits and lifestyle may affect the pharmacokinetics of drugs (eg, smoking, alcohol)

Yan Liu et al. reviewed published research for TDM over the past 30 years and summarized that the hotspots of TDM drugs in the last 5 years were vancomycin and medications related to inflammatory bowel disease (IBD) (Liu et al., 2022). In China, there has been a clinical practice guideline on vancomycin TDM since 2015, updated in 2020 (He et al., 2020), which has greatly promoted vancomycin TDM implementation. The proportion of hospitals implementing TDM for vancomycin is also higher (58.33%) than for many other drugs. For IBD therapy, monoclonal antibodies against tumor necrosis factor such as infliximab, adalimumab, certolizumab pegol and golimumab have all been approved worldwide for the treatment of moderate-to-severe active Crohn’s disease and/or ulcerative colitis (Argollo et al., 2020). Reactive TDM and proactive TDM have been proven as useful tools for optimizing biologic therapy, specifically anti-TNF therapy (Papamichael et al., 2019; Sparrow et al., 2020; Syed et al., 2020). While our data showed that no hospital conducted TDM for adalimumab, and only one hospital performed Infliximab TDM (data not shown). Furthermore, as the incidence of antimicrobial resistance has increased globally and the “average” human host has changed as well, except for antibiotics with narrow therapeutic windows such as vancomycin and aminoglycosides, TDM of beta-lactam antibiotics is also becoming necessary, especially for patients in ICU (Muller et al., 2018). While our results showed that only eight hospitals performed TDM of beta-lactam antibiotics (data not shown).

Overall, the clinical practice of TDM in China lagged behind the frontiers of TDM research in some fields. Confronted with this situation, staff in the responsible departments such as clinical pharmacists could form keeping-up groups. In this way, pharmacists track the cutting-edge TDM research, conduct promotion and education on drugs with the latest high-quality evidence-based research or published consortium guidelines that support conducting TDM. Furthermore, the standardized procedures of TDM and internal/external quality control for new projects should also be formulated in time to ensure the feasibility in clinical practice.

PharmGKB is a comprehensive resource that gathers knowledge about the impact of genetic variation on drug response for clinicians and researchers (PharmGKB, 2022a). According to PGx-based drug dosing guidelines annotates by the website, PGx testing at present could guide the clinical application of hundreds of drugs encompassing anticoagulant, anti-tumor, antiviral, anti-inflammatory, analgesic, anesthetic, anti-ulcer, anti-depression, anti-asthma and so on.

Based on the results of our survey and the information on Drug Label Annotations from PharmGKB (PharmGKB, 2022b), we matched the drugs conducted PGx testing with corresponding genes in Table 5. Clopidogrel and warfarin performed PGx testing in more than half of Chinese hospitals with PGx testing in our survey. Being an oral antiplatelet prodrug, clopidogrel is metabolized by *CYP2C19* gene into the active form. *CYP2C19* gene variants are known to be associated with increased or decreased response to clopidogrel (Wang et al., 2016). It has been demonstrated that patients with *CYP2C19* loss-of-function alleles have a higher risk of major adverse cardiovascular outcomes when treated with clopidogrel (Mega et al., 2010). Warfarin is a classic oral anticoagulant with a narrow therapeutic window and large inter-patient variability. About 53%–54% of dose variance of warfarin could be explained by taking into consideration both *VKORC1* and *CYP2C9* genetic polymorphisms (Gage et al., 2008). The guidelines published by the Clinical Pharmacogenetics Implementation Consortium (CPIC), the Canadian Pharmacogenomics Network for Drug Safety (CPNDS) and the Chinese Society of Cardiology all recommended that gene polymorphism testing of *CYP2C9* and *VKORC1* is beneficial to optimize the dosage regimen of Warfarin (Sun, 2013; Shaw et al., 2015; Johnson et al., 2017). PGx testing of clopidogrel and warfarin prior to initiating treatment is widely accepted and the clinical significance is generally recognized by clinicians.

**TABLE 5:** Drugs and corresponding Genes in PharmGKB.

Drug	Genes in drug labels				
	FDA	EMA	Swissmedic	HSCS	PMDA
Clopidogrel	*CYP2C19* ^d^	*CYP2C19* ^d^	*CYP2C19* ^d^	*CYP2C19* ^d^	*CYP2C19* ^d^
Warfarin	*CYP2C9* ^d^/*VKORC1* ^d^	/	/	*CYP2C9* ^d^/*VKORC1* ^d^	/
Statins^a^ (Atorvastatin)	*SLCO1B1* ^e^	/	/	/	/
Folic acid	/	/	/	/	/
Aspirin	/	/	*G6PD* ^d^	/	/
Allopurinol	*HLA-B* ^c^	/	*HLA-B* ^d^	/	*HLA-B* ^e^
Carbamazepine	*HLA-B* ^b^/*HLA-A* ^d^	/	*HLA-B* ^b^	*HLA-B* ^c^/*HLA-A* ^c^	*HLA-B* ^d^/*HLA-A* ^d^
			/*HLA-A* ^c^		
Irinotecan	*UGT1A1* ^*^ *28* ^d^	*UGT1A1* ^*^ *28* ^d^	*UGT1A1* ^*^ *28* ^d^	*UGT1A1* ^*^ *28* ^d^	*UGT1A1* ^c^
Tacrolimus	*CYP3A5* ^e^	/	/	/	/
Nitroglycerin	/	/	*G6PD* ^d^	/	/
Voriconazole	*CYP2C19* ^d^	*CYP2C19*/*CYP2C9*/*CYP3A4* ^e^	*CYP2C19* ^d^	*CYP2C19* ^d^	*CYP2C19* ^d^
Methotrexate	/	/	/	/	/
Glucocorticoid	/	/	/	/	/
Platinum	/	/	/	/	/
Ciclosporin	/	/	/	/	/

^a^ Atorvastatin is listed as a representative of stains. FDA: US, Food and Drug Administration; EMA: European Medicines Agency; Swissmedic: Swiss Agency of Therapeutic Products; HSCS: Health Canada (Santé Canada); PMDA: Pharmaceuticals and Medical Devices Agency, Japan. The human gene symbols have been italicized.

^b^ “Testing required”, refers to genetic testing, functional protein assays, cytogenetic studies, *etc.*, should be conducted before using this drug.

^c^ “Testing recommended”, refers to genetic testing, functional protein assays, cytogenetic studies, *etc.*, is recommended before using this drug.

^d^ “Actionable PGx”, refers to a gene, protein or chromosomal testing are not required or recommended.

^e^ “Informative PGx”, refers to particular variants or phenotypes affect a drug’s efficacy, dosage, metabolism or toxicity, but this effect is not “clinically” significant.

As compared with clopidogrel and warfarin, the conducting rate of PGx testing of other drugs in hospitals with PGx testing was relatively low, for instance, carbamazepine and allopurinol were both performed PGx testing in less than 20% of hospitals with PGx testing. However, it is necessary for patients to perform a PGx testing before using these drugs, for the severe adverse reactions maculopapular exanthem, Stevens-Johnson syndrome and toxic epidermal necrolysis are all related to Human leukocyte antigen B (*HLA-B*) genotype polymorphism (Saito et al., 2016; Phillips et al., 2018). One of the reasons limited PGx testing implementation of these drugs is the lack of approved reagent kits. On account of the user-friendliness and lower requirement for hardware environment when using PGx testing reagent kits, the number of approved kits could directly exert an impact on drugs tested. On the website of National Center for Clinical Laboratories and the National Medical Products Administration (National Medical Products Administration, 2022), the approved kits of clopidogrel and warfarin were 15 and seven respectively in 2019, which could meet the demand for clinical PGx testing well. On the contrary, only three kits were approved for PGx testing of the *HLA-B* gene.

Based on our results, recently, few PGx testing projects have successfully transformed from basic medical research into clinical practice. The latency of clinical implementation was contributed by many factors: the paucity of sufficiently powered trials that can quantify the added value of PGx testing in the real world (Lauschke and Ingelman-Sundberg, 2020); the negative attitude of physicians and the insufficient awareness of patients towards PGx (Guo et al., 2021); lack of approved reagent kits and equipment for conducting PGx testing; the shortage of qualified personnel (Owusu Obeng et al., 2018; Zhang J. et al., 2021; Tsuji et al., 2021); the high cost and unsound reimbursement policies, and so on. To promote the integration of basic research and clinical practice of PGx testing, it is significant to build good cooperation among the pharmaceutical industry, scientific research system and health system. The formulation of national policies and the innovation of testing technologies are also indispensable for the development of PGx transformation.

However, the utility of PGx testing in clinical practice should be viewed rationally and objectively. PGx testing results alone cannot determine medication regimens, even if the project is at a high level of evidence. For example, the TAILOR-PCI randomized clinical trial compared the genotype-guided selection of an oral *P2Y12* inhibitor with conventional clopidogrel therapy without point-of-care genotyping, resulting in no statistically significant difference in a composite endpoint of cardiovascular death (Pereira et al., 2020). Clinicians and pharmacists still need to combine individual pathophysiological status and other clinical indications to achieve precise medication.

### 4.5 The situation of PMC development in hospitals

As mentioned previously, the proportion of hospitals with PMC services in China was not satisfactory enough, and the development in different kinds of hospitals was unbalanced. Recently, the NHC has provided development proposals and created a promising environment to support the development of PMC in China. It is suggested that the implementation of PMC will be included in the evaluation index for hospital grade in the future.

The major types of PMC in China include independent PMC (specialized/general) and collaborative PMC. Our results showed that general PMC accounted for the largest proportion in all kinds of hospitals with PMC. Specialty PMCs include medication management for pregnancy/lactation, anticoagulant/antithrombotic, chronic disease management, pain management and so on, which are more suitable for patients with a single system disease. A general PMC refers to a pharmacist-managed clinic that does not differentiate specialties, being suitable for patients with multi-system diseases. Correspondingly, the requirements for pharmacists are much higher than specialty PMCs. The two types of PMC can complement each other in clinical practice. As for collaborative PMCs, pharmacists usually provide patients with pharmaceutical care together with physicians. This kind of pharmaceutical care might be relatively more easily accepted by patients. However, on account of the high dependence on physicians, only in hospitals with good physician-pharmacist partnerships can it be implemented, which also explains why this type of PMC was not common in our survey. Recently, online medical consultation services have been set up in more and more hospitals, and so does the online pharmaceutical care service. In this way, the timeliness and accessibility of high-quality medical care have been considerably improved, and the turnover can be reduced, which is meaningful during the COVID-19 pandemic (Wu et al., 2020). It was reported that most urological patients (84.7%) had a good acceptance of telemedical consultation (Boehm et al., 2020). Accordingly, it might be a practicable way for hospitals to establish online pharmaceutical care services in clinical practice.

In 2013, it was raised by the International Pharmaceutical Federation that pharmaceutical care without payment is not sustainable. This may lead to an inactive attitude of pharmaceutical practitioners, and the service quality cannot be well guaranteed. However, our survey showed that most PMCs in China were free of charge (only 21.08% were charged) and there is no uniform charge standard across China currently. In Japan, patients need to pay for the pharmacy information provision service and the medication management service, as well as in Korea. Policymakers should learn from these countries and enact the *Pharmacist Law* as soon as possible to help pharmacists increase enthusiasm for PMC service, which will also help with the translation of patients’ attitude towards PMC and improve the patients’ confidence in pharmacists.

Moreover, PMC is also an important way to bridge the gap between TDM or PGx testing and their clinical implementation. In October 2021, the NHC issued the standards for the pharmacist-managed clinic service in medical institutions, patients who require pharmacists to interpret the results of drug monitoring (e.g., blood drug concentration and PGx testing) are the targeted population for service (National Health Commission, 2021). Therefore, it is highly recommended that related departments integrate PMC, TDM and PGx testing services into an entirety, thereby spurring the development of individual pharmaceutical care.

### 4.6 Advantages and limitations

Our study for all we know is the first nationwide survey on individualized pharmaceutical care in China. That is, the biggest advantage of our survey is its large size, being able to represent the overall situation of individualized pharmaceutical care in China. And the quality of our data was well guaranteed by the support of the National Institute of Hospital Administration. Because our survey was conducted for pharmaceutical quality control and did not involve hospital-grade evaluation, the objectivity of the data was well guaranteed. And the items set in the e-questionnaire were mostly choice questions, which was also beneficial for improving the response rate and data reliability. It can be seen that the response rate is excellent. Furthermore, given that PMC is an indispensable part of individualized pharmaceutical care as well, it is quite meaningful to raise the awareness of PMC among medical practitioners. Our survey not only gathered data about TDM and PGx but paid attention to the status of PMC as well, which will be useful for policymakers to formulate strategies to promote further development of PMC.

There are also some limitations to our study. As mentioned above, a few hospitals filled the index of drugs and genes tested for PGx with diseases or irrelevant information, making it impossible to make sure whether a specific drug performed PGx testing in these hospitals or not. After careful consideration, we think that it was mainly because Part 2, question eight of the questionnaire (Supplementary material S2) was not clarified, contributing to some people’s misunderstanding concerning “PGx tests”. The fill-in-blank form with the indeterminate filling format is also one of the reasons leading to this problem. To cope with this situation, we excluded the non-standard data and outliers, ensuring the reliability and accuracy of our analyses as far as possible. Additionally, our test results were similar to concurrent studies in northern China (Zhang C. et al., 2021), so we conclude that there was no big influence on our test results. The National Institute of Hospital Administration should compile all test items based on the results of this survey and all reagent kits approved by the National Health Commission, match genes to drugs, present these questions as multiple choices when designing follow-up questionnaires.

In addition, our evaluation indexes were relatively not detailed enough. It was mainly because the scale of our study was too large to make a further survey on the premise of ensuring data accuracy and reliability. Furthermore, our study was a cross-sectional one and was the first study of this nature in China, so it was inevitable that actual patients’ longitudinal outcomes about TDM or PGx were unavailable. However, the National Institute of Hospital Administration will conduct regular research on individualized pharmaceutical care in the future. In this way, the prospective longitudinal data will be available and the trend of this field can be analyzed and discussed.

More studies focused on one field in individualized pharmaceutical care should be conducted for in-depth research. For example, researchers can also collect data such as the analytical methods and the implementation way for TDM service and PGx testing. As for PMC, further studies can be launched about the types of specialty PMC, the content of pharmaceutical care, education background and the degree of patient satisfaction, *etc.* Besides, more attention should be paid to the practice of addressing difficulties in the development of individualized pharmaceutical care. That is, sharing the practical experience of pilot hospitals with others should be encouraged and the construction of a curriculum system for cultivating professional pharmacists ought to be accelerated.

## 5 Conclusion

Overall, the development of individualized pharmaceutical care in China is still in the early stage. The implementation rate of TDM, PGx and PMC is relatively low, uniform clinical standards are deficient, the proportion of hospitals conducting EQC needs to be further improved, and the clinical practice in some fields lagged behind the cutting-edge research. Many different sectors and activities have to coalesce to promote the implementation and adoption of individualized pharmaceutical care, including the appropriate education, coverage, reimbursement policies, high-quality evidence, data systems, health system processes and health policies. To establish a completed and sophisticated individualized pharmaceutical care system, there is still a long way to go.

## Data Availability

The raw data supporting the conclusion of this article will be made available by the authors, without undue reservation.

## References

[B1] Ab Rahman A. F. Ahmed Abdelrahim H. E. Mohamed Ibrahim M. I. (2013). A survey of therapeutic drug monitoring services in Malaysia. Saudi Pharm. J. 21 (1), 19–24. 10.1016/j.jsps.2012.01.002 23960816 PMC3744941

[B2] Alrabiah Z. Alwhaibi A. Alsanea S. Alanazi F. K. Abou-Auda H. S. (2021). A national survey of attitudes and practices of physicians relating to therapeutic drug monitoring and clinical pharmacokinetic service: Strategies for enhancing patient's care in Saudi arabia. Int. J. Gen. Med. 14, 1513–1524. 10.2147/ijgm.S296731 33935513 PMC8079248

[B3] Argollo M. Kotze P. G. Kakkadasam P. D'Haens G. (2020). Optimizing biologic therapy in IBD: How essential is therapeutic drug monitoring? Nat. Rev. Gastroenterol. Hepatol. 17 (11), 702–710. 10.1038/s41575-020-0352-2 32879465

[B4] Ballotari M. Taus F. Tolle G. Danese E. Dorizzi R. M. Tagliaro F. (2022). Development of a new ultra-high-performance liquid chromatography-tandem mass spectrometry method for the determination of digoxin and digitoxin in plasma: Comparison with a clinical immunoassay. Electrophoresis 43 (9-10), 1019–1026. 10.1002/elps.202100290 35132652 PMC9303718

[B5] Boehm K. Ziewers S. Brandt M. P. Sparwasser P. Haack M. Willems F. (2020). Telemedicine online visits in urology during the COVID-19 pandemic-potential, risk factors, and patients' perspective. Eur. Urol. 78 (1), 16–20. 10.1016/j.eururo.2020.04.055 32362498 PMC7183955

[B6] Chen N. Zhang X. Gong Y. Kong L. (2015). Analysis and prospect of hospital pharmacy department to carry out the individualized pharmaceutical care. Chin. Hosp. Pharm. J. 35 (15), 1343–1346. 10.13286/j.cnki.chinhosppharmacyj.2015.15.01

[B7] Choi R. Woo H. I. Park H. D. Lee S. Y. (2019). A nationwide utilization survey of therapeutic drug monitoring for five antibiotics in South Korea. Infect. Drug Resist 12, 2163–2173. 10.2147/idr.S208783 31410036 PMC6646174

[B8] Deprez S. Stove C. P. (2021). Fully automated dried blood spot extraction coupled to liquid chromatography-tandem mass spectrometry for therapeutic drug monitoring of immunosuppressants. J. Chromatogr. A 1653, 462430. 10.1016/j.chroma.2021.462430 34384960

[B9] Division of Therapeutic Drug Monitoring C.P.S.Committee C.P.A.M.B.A.P.Sciences D.I.o.C.P.C.A.o. (2021). Consensus on the use of chromatography for quality assurance of therapeutic drug monitoring (2021 edition). Chin. Pharm. J. 56 (17), 1443–1448.

[B10] National Center for clinical Laboratories Beijing CHAO-YANG hospital, c.m.u. Dong C. f. c. L. i. G. (2013). Guidelines for the application of external quality assessment results. Beijing: National Health Commission of the People's Republic of China.

[B11] Gage B. F. Eby C. Johnson J. A. Deych E. Rieder M. J. Ridker P. M. (2008). Use of pharmacogenetic and clinical factors to predict the therapeutic dose of warfarin. Clin. Pharmacol. Ther. 84 (3), 326–331. 10.1038/clpt.2008.10 18305455 PMC2683977

[B12] Guo C. Hu B. Guo C. Meng X. Kuang Y. Huang L. (2021). A survey of pharmacogenomics testing among physicians, pharmacists, and researchers from China. Front. Pharmacol. 12, 682020. 10.3389/fphar.2021.682020 34322018 PMC8311355

[B13] He N. Su S. Ye Z. Du G. He B. Li D. (2020). Evidence-based guideline for therapeutic drug monitoring of vancomycin: 2020 update by the division of therapeutic drug monitoring, Chinese pharmacological society. Clin. Infect. Dis. 71 (4), S363–s371. 10.1093/cid/ciaa1536 33367582

[B14] Hu X. Jia T. Zhang X. Wu C. Zhang Y. Chen J. (2022). Clinical pharmacists' involvement in pharmacogenomics testing and related services in China. J. Pers. Med. 12 (8), 1267. 10.3390/jpm12081267 36013216 PMC9409798

[B15] Jameson J. L. Longo D. L. (2015). Precision medicine--personalized, problematic, and promising. N. Engl. J. Med. 372 (23), 2229–2234. 10.1056/NEJMsb1503104 26014593

[B16] Johnson J. A. Caudle K. E. Gong L. Whirl-Carrillo M. Stein C. M. Scott S. A. (2017). Clinical pharmacogenetics implementation consortium (CPIC) guideline for pharmacogenetics-guided warfarin dosing: 2017 update. Clin. Pharmacol. Ther. 102 (3), 397–404. 10.1002/cpt.668 28198005 PMC5546947

[B17] Kisor D. F. Calinski D. M. Farrell C. L. (2018). Beyond the didactic lecture: Pharmacogenomics in pharmacy education. Per Med. 15 (1), 9–12. 10.2217/pme-2017-0056 29714112

[B18] Lauschke V. M. Ingelman-Sundberg M. (2020). Emerging strategies to bridge the gap between pharmacogenomic research and its clinical implementation. NPJ Genom Med. 5, 9. 10.1038/s41525-020-0119-2 32194983 PMC7057970

[B20] Liu K. Huang H. Zhang L. Huang Y. Sun S. Chen X. (2021). Effects of a physician- and pharmacist-managed clinic on pain management in cancer patients in China. Basic Clin. Pharmacol. Toxicol. 129 (1), 36–43. 10.1111/bcpt.13583 33763950

[B21] Liu Y. Liu D. Huang L. Zeng L. Zhang L. (2022). Trends and hotspots in therapeutic drug monitoring over the last 30 years: Visualization and analysis based on CiteSpace. Chin. J. Hosp. Pharm., 1–11. 10.13286/j.1001-5213.2022.12.06

[B22] Marcinak R. Paris M. Kinney S. R. M. (2018). Pharmacogenomics education improves pharmacy student perceptions of their abilities and roles in its use. Am. J. Pharm. Educ. 82 (9), 6424. 10.5688/ajpe6424 30559496 PMC6291667

[B23] Matey E. T. Ragan A. K. Oyen L. J. Vitek C. R. Aoudia S. L. Ragab A. K. (2022). Nine-gene pharmacogenomics profile service: The Mayo Clinic experience. Pharmacogenomics J. 22 (1), 69–74. 10.1038/s41397-021-00258-0 34671112

[B24] Mayo Clinic (2019). Pharmacogenomics for your practice – online CME course [online]. Available: https://ce.mayo.edu/online-education/content/pharmacogenomics-your-practice-%E2%80%93-online-cme-course (Accessed 08 13, 2022).

[B25] Mega J. L. Simon T. Collet J. P. Anderson J. L. Antman E. M. Bliden K. (2010). Reduced-function CYP2C19 genotype and risk of adverse clinical outcomes among patients treated with clopidogrel predominantly for PCI: A meta-analysis. Jama 304 (16), 1821–1830. 10.1001/jama.2010.1543 20978260 PMC3048820

[B26] Muller A. E. Huttner B. Huttner A. (2018). Therapeutic drug monitoring of beta-lactams and other antibiotics in the intensive care unit: Which agents, which patients and which infections? Drugs 78 (4), 439–451. 10.1007/s40265-018-0880-z 29476349

[B27] National Center for Clinical Laboratories (2022). Available: https://www.nccl.org.cn/mainCn (Accessed 0814, 2022).

[B28] National Clinical Improvement System. (2019). Available: https://ncisdc.medidata.cn/login.jsp (Accessed 0813, 2022).

[B29] National Health and Family Planning Commission (2015). Guidelines for genetic testing of drug metabolism enzymes and drug action targets (trial edition). Pract. J. Organ Transplantation(Electronic Version) 3 (05), 257–267.

[B30] National Health Commission (2021). Notice on the issuance of 5 standards for pharmacist-managed clinic services and so on in medical institutions [Online]. Available: http://www.nhc.gov.cn/yzygj/s7659/202110/f76fc77acd87458f950c86d7bc468f22.shtml (Accessed May 17, 2022).

[B31] National Health Commission (2018). Opinions on accelerating the high-quality development of pharmaceutical care [Online]. Available: http://www.gov.cn/xinwen/2018-11/28/content_5344128.htm (Accessed 0215, 2023).

[B32] National Medical Products Administration (2022). Available: https://www.nmpa.gov.cn/ (Accessed 0814, 2022).

[B33] Owusu Obeng A. Fei K. Levy K. D. Elsey A. R. Pollin T. I. Ramirez A. H. (2018). Physician-reported benefits and barriers to clinical implementation of genomic medicine: A multi-site IGNITE-network survey. J. Pers. Med. 8 (3), 24. 10.3390/jpm8030024 30042363 PMC6163471

[B34] Papamichael K. Vogelzang E. H. Lambert J. Wolbink G. Cheifetz A. S. (2019). Therapeutic drug monitoring with biologic agents in immune mediated inflammatory diseases. Expert Rev. Clin. Immunol. 15 (8), 837–848. 10.1080/1744666x.2019.1630273 31180729

[B35] Patsalos P. N. Spencer E. P. Berry D. J. (2018). Therapeutic drug monitoring of antiepileptic drugs in epilepsy: A 2018 update. Ther. Drug Monit. 40 (5), 526–548. 10.1097/ftd.0000000000000546 29957667

[B36] Pedersen C. A. Schneider P. J. Scheckelhoff D. J. (2016). ASHP national survey of pharmacy practice in hospital settings: Monitoring and patient education-2015. Am. J. Health Syst. Pharm. 73 (17), 1307–1330. 10.2146/ajhp160081 27413141

[B37] Pereira N. L. Farkouh M. E. So D. Lennon R. Geller N. Mathew V. (2020). Effect of genotype-guided oral P2Y12 inhibitor selection vs conventional clopidogrel therapy on ischemic outcomes after percutaneous coronary intervention: The TAILOR-PCI randomized clinical trial. Jama 324 (8), 761–771. 10.1001/jama.2020.12443 32840598 PMC7448831

[B38] PharmGKB (2022b). Available: https://www.pharmgkb. org/ (Accessed 0813, 2022).

[B39] PharmGKB (2022a). Drug Label Annotations [online]. Available: https://www.pharmgkb.org/labelAnnotations (Accessed May 17, 2022).

[B40] Phillips E. J. Sukasem C. Whirl-Carrillo M. Müller D. J. Dunnenberger H. M. Chantratita W. (2018). Clinical pharmacogenetics implementation consortium guideline for HLA genotype and use of carbamazepine and oxcarbazepine: 2017 update. Clin. Pharmacol. Ther. 103 (4), 574–581. 10.1002/cpt.1004 29392710 PMC5847474

[B41] Saito Y. Stamp L. K. Caudle K. E. Hershfield M. S. McDonagh E. M. Callaghan J. T. (2016). Clinical pharmacogenetics implementation consortium (CPIC) guidelines for human leukocyte antigen B (HLA-B) genotype and allopurinol dosing: 2015 update. Clin. Pharmacol. Ther. 99 (1), 36–37. 10.1002/cpt.161 26094938 PMC4675696

[B42] Shaw K. Amstutz U. Kim R. B. Lesko L. J. Turgeon J. Michaud V. (2015). Clinical practice recommendations on genetic testing of CYP2C9 and VKORC1 variants in warfarin therapy. Ther. Drug Monit. 37 (4), 428–436. 10.1097/ftd.0000000000000192 26186657

[B43] Sparrow M. P. Papamichael K. Ward M. G. Riviere P. Laharie D. Paul S. (2020). Therapeutic drug monitoring of biologics during induction to prevent primary non-response. J. Crohns Colitis 14 (4), 542–556. 10.1093/ecco-jcc/jjz162 31549158 PMC7392326

[B44] Sun Y. (2013). Expert consensus on warfarin anticoagulant therapy in China. Chin. J. Intern Med. 52 (01), 76–82.

[B45] Syed N. Tolaymat M. Brown S. A. Sivasailam B. Cross R. K. (2020). Proactive drug monitoring is associated with higher persistence to infliximab and adalimumab treatment and lower healthcare utilization compared with reactive and clinical monitoring. Crohns Colitis 360 (3), otaa050. otaa050. 10.1093/crocol/otaa050 PMC7380488 32743546

[B46] Tsuji D. Saito Y. Mushiroda T. Miura M. Hira D. Terada T. (2021). Results of a nationwide survey of Japanese pharmacists regarding the application of pharmacogenomic testing in precision medicine. J. Clin. Pharm. Ther. 46 (3), 649–657. 10.1111/jcpt.13367 33555613

[B47] University of Florida Health (2022). Pharmacogenomics and precision medicine online graduate &professional program [online]. Available: https://onlinepm.pharmacy.ufl.edu/?gclid=EAIaIQobChMIwqiKneqL6gIVE9bACh2-1wUyEAAYAiAAEgLsNPD_BwE (Accessed 0813, 2022).

[B48] Vo T. T. Bell G. C. Owusu Obeng A. Hicks J. K. Dunnenberger H. M. (2017). Pharmacogenomics implementation: Considerations for selecting a reference laboratory. Pharmacotherapy 37 (9), 1014–1022. 10.1002/phar.1985 28699700

[B49] Wang Y. Zhao X. Lin J. Li H. Johnston S. C. Lin Y. (2016). Association between CYP2C19 loss-of-function allele status and efficacy of clopidogrel for risk reduction among patients with minor stroke or transient ischemic attack. Jama 316 (1), 70–78. 10.1001/jama.2016.8662 27348249

[B50] Wenxiang C. (2013). External quality assurance in laboratory medicine: A status report. Chin. J. Clin. Laboratory Manag. Electron. Ed. 1 (01), 8–11.

[B51] Wu H. Sun W. Huang X. Yu S. Wang H. Bi X. (2020). Online antenatal care during the COVID-19 pandemic: Opportunities and challenges. J. Med. Internet Res. 22 (7), e19916. 10.2196/19916 32658860 PMC7407486

[B52] Zhang C. Lei J. Liu Y. Wang Y. Huang L. Feng Y. (2021a). Therapeutic drug monitoring and pharmacogenetic testing in northern China. Front. Pharmacol. 12, 754380. 10.3389/fphar.2021.754380 34795589 PMC8593476

[B53] Zhang J. Qi G. Han C. Zhou Y. Yang Y. Wang X. (2021b). The landscape of clinical implementation of pharmacogenetic testing in central China: A single-center study. Pharmgenomics Pers. Med. 14, 1619–1628. 10.2147/pgpm.S338198 34934339 PMC8684419

[B54] Zhang X. Miao L. Chen W. (2019). The expert consensus on the standards of therapeutic drug monitoring. Eval. analysis drug-use Hosp. China 19(08), 897–898. 10.14009/j.issn.1672-2124.2019.08.001

