# Supplementary figures and images for: A national survey of individualized pharmaceutical care practice in Chinese hospitals in 2019
Source: Front Pharmacol. 2023 Mar 2;14:1022134. doi: 10.3389/fphar.2023.1022134 (PMC10018172; doi:10.3389/fphar.2023.1022134)

## The data collection work guideline for medical institutions

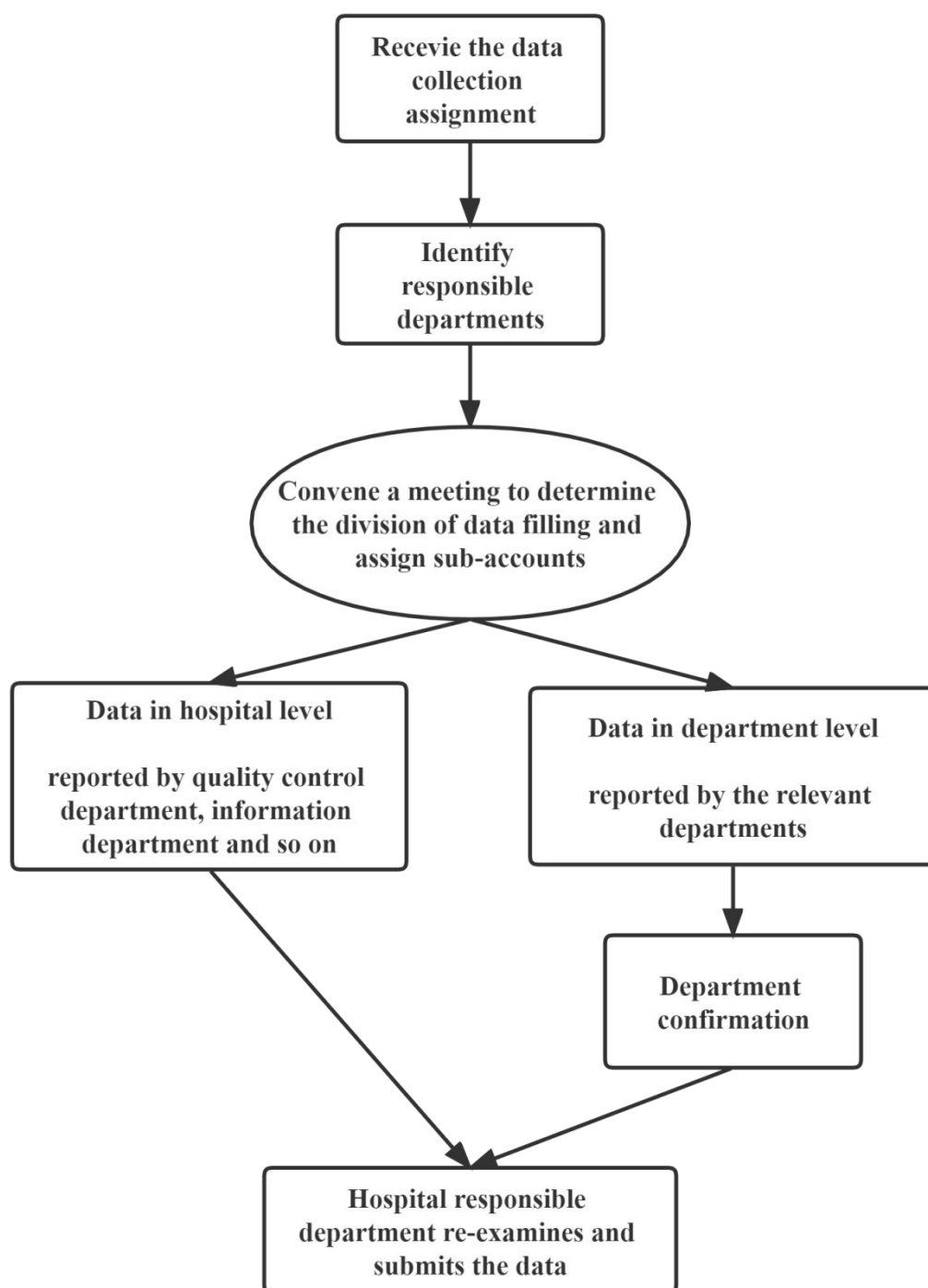

Supplement: Supplementary file 1 [file Presentation1.pdf]
