# Supplementary material for: A national survey of individualized pharmaceutical care practice in Chinese hospitals in 2019
Source: Front Pharmacol. 2023 Mar 2;14:1022134. doi: 10.3389/fphar.2023.1022134 (PMC10018172; doi:10.3389/fphar.2023.1022134)
Supplement: Supplementary file 2 [file Presentation2.pdf]

Questionnaire of Individualized Pharmaceutical Care

Excerpt from the questionnaire of Medical Quality Management and Control in 2019

We are the National Institute of Hospital Administration, Beijing. This nationwide survey aims to enhance pharmaceutical administration and quality control in medical institutions. Please fill in the questionnaire carefully according to the actual situation in your medical institution. This questionnaire's data quality, accuracy, and completeness will be used as a benchmark for hospital evaluation and key specialty settings. We shall take appropriate action to safeguard the security of the data.

If you have any questions or comments regarding this questionnaire, kindly get in touch with us using the information shown in the filling system.

Please note:
1. *If the hospital has not provided a given service, or has provided the service while the corresponding data has not been counted or cannot be counted, please enter "/", rather than "0", as the value "0" has statistical significance!*
2. *We gather the data range from January 1, 2019 to December 31, 2019.*

The confirmation of hospital's basic information

1. The name of your medical institution[fill in the blank]: _________________
2. The registration number of practice license of your medical institution[fill in the blank]: __________________
3. Which province/autonomous region/municipality do your medical institution locate in?

○ Beijing ○ Tianjin ○ Shanghai ○ Hainan ○ Heilongjiang ○ Jilin ○ Liaoning
○ Neimenggu ○ Ningxia ○ Xinjiang ○ Xizang ○ Qinghai ○ Gansu ○ Zhejiang
○ Shanxi ○ Anhui ○ Chongqing ○ Guangxi ○ Guizhou ○ Hebei ○ Jiangxi ○ Shaanxi
○ Yunan ○ Fujian ○ Guangdon ○ Henan ○ Hubei ○ Hunan ○ Jiangsu ○ Shandong
○ Sichuan

4. Which city do your medical institution locate in[fill in the blank]? ___________
5. The ownership of your medical institution is?

○Public
○Private

6. The type of your medical institution is？

○General
○Specialty

7. The grade of your medical institution is?

○Tertiary (best)
○Secondary
○Grade-I

8. The level of your medical institution is?
○A (best)
○B
○C

Part 1 The implementing situation of Therapeutic drug monitoring (TDM)
1. Does your medical institution provide TDM?
○YES
○NO (skip to the next part)
2. What is the responsible department of TDM?
○Pharmacy department
○Clinical laboratory
○Multi-department
○Others_______
○Unknown
3. Does your medical institution conduct external quality control for TDM?
○YES
○NO
○Unknown
4. Please list all drugs that conducted therapeutic drug monitoring in your medical institution (in 200 words)[fill in the blank].
____.

Part 2 The implementing situation of Pharmacogenomic testing
Pharmacogenomic (PGx) testing: a test to identify target genes or drug metabolism in order to determine the causes of adverse drug reactions or poor drug efficacy and to help choose the therapeutic pharmaceuticals and their dosages.

5. Does your medical institution provide PGx testing?
○YES
○NO (skip to the next part)
6. What is the responsible department of PGx testing?
○Pharmacy department
○Pathology department
○Clinical laboratory
○Multi-department
○Others________
○Unknown
7. Does your medical institution conduct external quality control for PGx testing?
○YES
○NO
○Unknown
8. Please list all PGx tests in clinical practice in your medical institution (in 200 words)[fill in the blank].
____.

Part 3 The situation of Pharmacist-Managed Clinic

Pharmacist-Managed Clinic (PMC) refers to a pharmaceutical outpatient clinic where patients could get professional medication guidance after registering through the hospital registration system. It includes multidisciplinary collaborative PMC and independent PMC. The medication consultations in the drug consultation window or consultation room are excluded.
(1) Collaborative PMC refer to a service mode in which pharmacists provide pharmaceutical care for patients together with doctors or personnel from other departments;
(2) Independent PMC refer to a service mode in which pharmacists provide pharmaceutical care independently. Independent PMC includes specialized clinics and general clinics.

9. Does your medical institution provide Pharmacist-Managed Clinic?
○YES
○NO (skip to the next part)
10. What types of PMC are in your medical institutions?
○Collaborative PMC (skip to Q12)
○Independent PMC
○More than one type
○Unknown (skip to Q12)
11. The independent PMC in your medical institution is
○specialized PMC
○general PMC
○specialized and general PMCs
12. Does the pharmacist-managed clinic charge in your medical institution?
○YES
○NO

Supplementary information
1. Name: ___________
2. Clinical Department: __________
3. Professional title: __________
4. Phone number: ___________
5. If there is anything that needs to be explained, please fill in here.

____

Declaration: I can give a tracking check and the information provided in this survey is accurate and reliable.
